# Identification of Fecal Microbiota and Related Metabolites Associated with Feed Efficiency in DLY Pigs

**DOI:** 10.3390/ani15203026

**Published:** 2025-10-18

**Authors:** Zhicheng Zhang, Kuirong Chen, Shuai Zhang, Yiyun He, Guofeng Lei, Yunxiang Zhao, Jing Liang

**Affiliations:** College of Animal Science & Technology, Guangxi University, Nanning 530004, China; 18775085095@163.com (Z.Z.); 15737555093@163.com (K.C.); zs714096718@163.com (S.Z.); m15708893213@163.com (Y.H.); 15885966834@163.com (G.L.)

**Keywords:** DLY pigs, feed efficiency, fecal microbiota, gut metabolites, steroid hormones, bile acids

## Abstract

**Simple Summary:**

Improving feed efficiency (FE) is crucial for modern pig breeding due to its significant economic and environmental impacts. Fecal microbiota play a critical role in this by synthesizing various beneficial substances that enhance FE. This study utilized 16S rRNA gene sequencing and LC-MS to analyze microbial and metabolic differences between pigs with high and low feed conversion ratios (FCR). Results indicated that pigs with lower FCR had an increased abundance of beneficial SCFA-producing bacteria, while higher-FCR pigs exhibited greater levels of pathogenic taxa. These findings highlight microbial and metabolic contributions to feed efficiency and provide insights into targeted breeding strategies.

**Abstract:**

Improving feed efficiency (FE) is essential for enhancing productivity, reducing production costs, and minimizing environmental impacts in the swine industry. Fecal microbiota and their metabolites play important roles in nutrient metabolism and energy utilization. This study aimed to investigate the fecal microbiota and associated metabolites in pigs with divergent feed conversion ratios (FCR). Fecal samples were collected from 20 Duroc × (Landrace × Yorkshire) (DLY) commercial pigs exhibiting extremely high (HFCR, *n* = 10) and low (LFCR, *n* = 10) FCR for analysis using 16S rRNA gene sequencing and liquid chromatography–mass spectrometry (LC-MS). The microbiota analysis revealed significantly higher abundances of *Ruminococcus*, *Prevotella*, *Akkermansia*, and *Eubacterium* in LFCR pigs (*p* < 0.05), while pathogenic bacteria predominated in HFCR pigs (*p* < 0.05). LC-MS metabolomics identified significant variations in metabolites involved in steroid hormone biosynthesis and primary bile acid metabolism between the two groups (*p* < 0.05). Spearman correlation analysis further demonstrated significant positive correlations between *Ruminococcaceae_NK4A214_group* and *[Eubacterium]_coprostanogenes_group* with bile acid metabolites, as well as between *Akkermansia* and steroid hormone synthesis (*p* < 0.05). These findings suggest a potential role for specific microbes and metabolites that are associated with feed efficiency, and warrant validation in pig feeding trials and fecal microbiota transplantation (FMT).

## 1. Introduction

Pork constitutes the principal meat consumed worldwide and is a significant source of high-quality protein and key micronutrients [1]. As the global population expands and household incomes continue to rise, worldwide pork consumption is projected to double by 2050 [2]. Therefore, finding ways to enhance pork production and fulfill human needs has become a pressing challenge in modern animal agriculture.

Feed costs account for approximately 60% of the total production expenses in pig farming, making FE a critical determinant of economic sustainability and profitability [3]. The main indices for evaluating FE are residual feed intake (RFI) and FCR [4]. RFI represents the difference between actual feed intake and expected feed intake [5], while FCR is defined as the ratio of average daily feed intake (ADFI) to average daily gain (ADG) [6]. The adoption of automated feeding systems has greatly improved the precision and convenience of monitoring these metrics, thereby facilitating data-driven selection in pig breeding programs [7].

FE is influenced by a combination of genetic, physiological, and environmental factors, with increasing evidence highlighting the role of fecal microbiota in modulating feed utilization [8]. These microbial communities are capable of fermenting complex carbohydrates, such as cellulose and resistant starches, which are otherwise indigestible by the host. This fermentation process produces short-chain fatty acids (SCFAs), which serve as energy sources for the host [9]. Previous studies have reported microbial taxa associated with divergent feed efficiency, such as the enrichment of Lachnospiraceae and Prevotellaceae, together with *Streptococcus* (notably *Streptococcus gallolyticus* subsp. *gallolyticus*), in high-FE DLY pigs [10], and enrichment of *Prevotella* CAG:604 in the cecum of pigs with high FCR (HFCR) [11]. Similarly, McCormack et al. found that an augmentation of specific fecal microbiota in pigs with low RFI was associated with improved health and FE [12]. These findings underscore the importance of fecal microbiota in shaping host metabolic performance and feed conversion.

Although fecal metabolomics offers valuable insights into microbial metabolic activity and host nutrient utilization, its application in feed efficiency studies remains limited [13,14]. Fecal metabolites, as the end products of host-microbe interactions, reflect both microbial composition and gut physiological processes. They serve as functional biomarkers for investigating the links between microbiota metabolism and host phenotypes [15]. Metabolomic analysis enables the identification of differential metabolites, offering a deeper understanding of metabolic pathways involved in nutrient absorption and energy balance. This integrative approach may support the development of targeted strategies to enhance FE in pig production.

Despite growing interest, the combined effects of fecal microbiota composition and fecal metabolites on FE remain inadequately understood. In this study, we employed 16S rRNA gene sequencing and liquid chromatography–mass spectrometry (LC/MS) to profile the fecal microbiota and metabolites in pigs with contrasting FCR. By integrating microbiome and metabolome data, we aimed to uncover key microbial and metabolic signatures associated with feed efficiency, thereby providing new targets for precision breeding and nutritional interventions in swine production.

## 2. Materials and Methods

### 2.1. Animal Management, Phenotypic Data Collection, and Sampling

A total of 384 Duroc × (Landrace × Yorkshire) (DLY) pigs were raised under uniform conditions on a commercial farm in Nanning, Guangxi, China. Individual feed intake and body weight were monitored from 90 to 165 days of age using an automatic feeding system (Nedap N.V., Groenlo, The Netherlands). ADFI, ADG, and FCR (FCR = ADFI/ADG) were calculated. Based on FCR values, 10 pigs with the highest FCR values as the HFCR group, and 10 pigs with the lowest FCR values as the LFCR group, were selected for further study. All pigs received the same diet ad libitum, had free access to water, remained clinically healthy, and were not treated with antibiotics during the trial. The ingredient composition and nutrient levels of the experimental diet are provided in Supplementary Table S1. At 170 days of age, fresh fecal samples were collected from all 384 pigs on the same day, immediately frozen in liquid nitrogen, transported on dry ice, and stored at −80 °C until analysis. Detailed growth performance for the HFCR and LFCR groups is provided in Supplementary Table S2, and the growth data for all 384 pigs are provided in Supplementary Table S3.

### 2.2. Fecal Microbiota Analysis

Total genomic DNA was extracted from fecal samples using the QIAamp Fast DNA Fecal Kit (Qiagen, Hilden, Germany). Negative controls were included during DNA extraction and subsequent PCR amplification to monitor for contamination. DNA concentration and integrity were assessed via Qubit fluorometry and 1% agarose gel electrophoresis. The V3–V4 region of the 16S rRNA gene was amplified using primers 341F (5′-CCTACGGGNGGWGCAG-3′) and 806R (5′-GACTACHVGGGTWTCTAAT-3′) with barcodes. PCR products with expected size and concentration (≥2 ng/μL) were purified and used to construct sequencing libraries. Libraries were quantified (≥4 nM), pooled, and sequenced on an Illumina MiSeq platform using the PE250 strategy. Amplification sub-process analysis: Bioinformatics analysis of the amplicons was conducted using EasyAmplicon (v1.0). After quality control of the original Illumina fastq file (with an error rate < 1%), redundancy was removed using VSEARCH with the parameter min_Unique_Size set to 59 [16]. Usearch (v11) was used to perform a 100% similarity comparison of ASVs, followed by the removal of plastids and non-bacterial sequences using the sintax_Cutoff parameter set to 0.1. Low-abundance ASVs were filtered using Vegan (v2.6.4) packages, and all samples were normalized to 99,888 sequences to determine ASV abundance. Species annotation was performed using the SILVA database (v138), and the results were used for amplicon analysis [17]. Alpha diversity indices (Richness, Chao1, ACE, Shannon, Simpson) were calculated using the Vegan package. Beta diversity was analyzed by Bray–Curtis distance-based Principal Coordinate Analysis (PCoA). Taxonomic composition was visualized at the phylum and family levels, and LEfSe (http://www.bic.ac.cn/BIC/, accessed on 15 March 2024) was used to identify differentially abundant genera (LDA > 3).

### 2.3. Fecal Metabolomics Analysis

Take the sample from the −80 °C refrigerator and thaw it on ice (all subsequent operations were performed on ice). Weigh 20 mg (±1 mg) of feces and transfer it to the appropriately labeled centrifuge tube. Add 400 µL of 70% methanol-water internal standard extraction solution and vortex for 3 min. If the sample is not dispersed, add steel balls and vortex for an additional 3 min. Sonicate in an ice-water bath for 10 min, then remove the sample and vortex for 1 min. Next, place the sample in a −20 °C freezer for 30 min. Centrifuge at 12,000× *g* for 10 min at 4 °C, and transfer 300 μL of the supernatant to a fresh microcentrifuge tube. Then, centrifuge at 12,000× *g* for 3 min and extract 200 µL of the supernatant for subsequent machine analysis [18]. Supernatants were used for LC-MS analysis. Samples were analyzed using a Triple TOF-6600 mass spectrometer (AB Sciex, Framingham, MA, USA) in both positive and negative ion modes. Chromatographic separation was performed on an LC20 UHPLC system (Shimadzu, Kyoto, Japan) with a Waters ACQUITY UPLC HSS T3 column. Mobile phases consisted of water with 0.1% formic acid (A) and acetonitrile with 0.1% formic acid (B).

Raw data were converted to mzXML using ProteoWizard and processed with XCMS for peak detection and alignment. Peaks with >50% missing values were excluded. After calibration and filtering, a metabolic spectrum table was compiled by querying the proprietary database (Wuhan Maiwei Metabolic Biotechnology Co., Ltd., Wuhan, China). Quality control was assessed using total ion current (TIC) overlays from pooled QC samples (Supplementary Figure S1). Normalized data were subjected to orthogonal partial least squares discriminant analysis (OPLS-DA) in SIMCA-P (version 14.1). Differential metabolites were identified using VIP > 1, *p* < 0.05, and FC > 2 or < 0.5. Annotated metabolites were mapped to the Human Metabolome Database (HMDB) and analyzed using MetaboAnalyst 5.0 and KEGG for pathway enrichment (Supplementary Table S4).

### 2.4. Statistical Analysis

Phenotypic traits were analyzed using SPSS version 26.0. Results are expressed as mean ± standard deviation (SD), and 95% confidence intervals (CI) are also reported to indicate the precision of estimates. Differences in alpha diversity and metabolite levels between groups were assessed using two-sided independent-samples *t*-tests with significance set at *p* < 0.05. Pairwise Spearman correlations (|ρ| > 0.6, *p* < 0.05) were computed among phenotypic traits, differential taxa, and metabolites to construct association networks.

## 3. Results

### 3.1. Phenotypic Differences Between HFCR and LFCR Pigs

Significant differences in growth traits were observed between HFCR and LFCR groups. Pigs in the LFCR group exhibited a significantly higher ADG, along with lower ADFI and FCR compared to those in the HFCR group (*p* < 0.001; Table 1).

### 3.2. Fecal Microbiota Composition Differences Between HFCR and LFCR Pigs

Alpha diversity metrics (Richness, Chao1, ACE) were significantly higher in LFCR pigs compared to HFCR pigs (*p* < 0.01), indicating greater microbial abundance. The Shannon index was also higher in LFCR pigs, though the difference was marginally significant (*p* = 0.05; Table 2).

**Figure 1:**
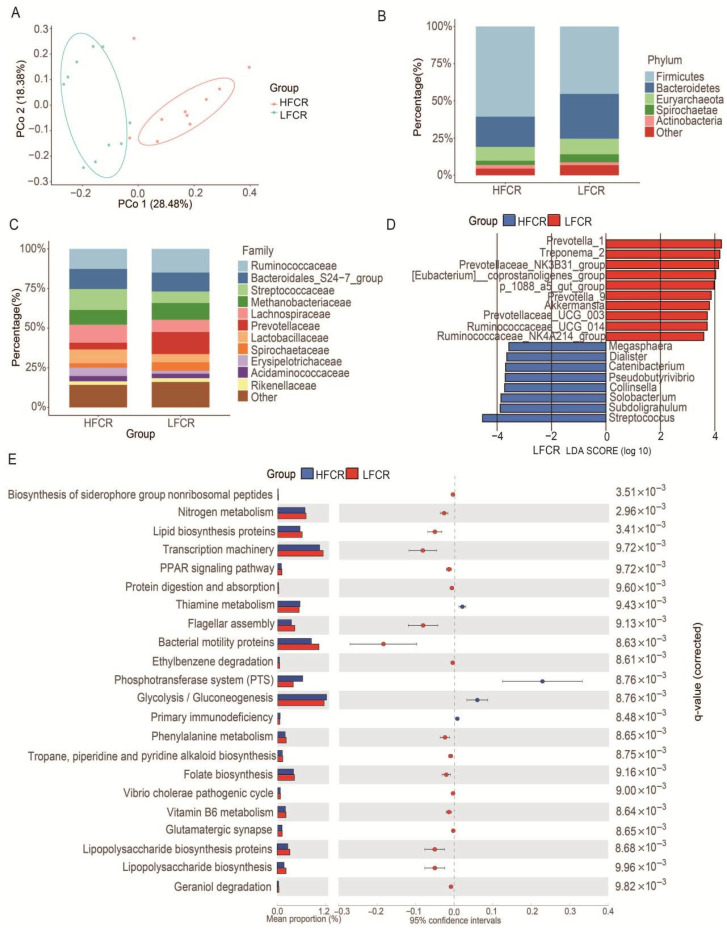
Fecal microbiota profiles in pigs with high and low feed conversion ratios (FCR). (**A**) Principal coordinates analysis (PCoA) based on Bray–Curtis distance showing distinct microbial community structures between HFCR (red) and LFCR (blue) groups. (**B**) Taxonomic composition of LFCR and HFCR groups at the phylum level. (**C**) Taxonomic composition of LFCR and HFCR groups at the family level. (**D**) LefSe analysis identifying differentially abundant genera (LDA score > 3) between groups. (**E**) Predicted functional pathways of microbial communities using PICRUSt and STAMP, with significant differences assessed by Welch’s *t*-test (*q* < 0.05). Relative abundances are expressed as group means. Blue and red denote HFCR and LFCR groups, respectively.

Principal Coordinate Analysis (PCoA) based on Bray–Curtis distances showed distinct microbial community structures between groups (PC1 = 28.48%, PC2 = 18.38%; Figure 1A). At the phylum level, Firmicutes and Bacteroidetes dominated both groups, with LFCR pigs showing higher Bacteroidetes and lower Firmicutes abundance (Figure 1B). At the family level, the LFCR group shows higher abundances of *Ruminococcaceae* and *Prevotellaceae* and lower abundances of *Streptococcaceae* and *Lachnospiraceae* than the HFCR group (Figure 1C). For the genus-level microbiota, genera with an average relative abundance greater than 0.5% were selected for LefSe analysis (LDA > 3; Figure 1D). LFCR-enriched genera included *Prevotella_1*, *Treponema_2*, *Prevotelaceae_NK3B31_group*, *[Eubacterium]_coprostanogenes_group*, *p_1088_a5_gut_group*, *Prevotella_9*, *Akkermansia*, *Prevotelaceae_UCG-003*, *Ruminococcaceae_UCG-014*, *Ruminococcaceae_NK4A214_group*. HFCR-enriched genera included *Megaspaera*, *Dialist*, *Catenibacterium*, *Pseudobotyrivibrio*, *Collinsella*, *Solobacterium*, *Subdoligranulum*, and *Streptococcus*. PICRUSt-based functional predictions revealed enhanced nitrogen metabolism and lipid biosynthesis potential in the LFCR group, whereas HFCR pigs had stronger functions in thiamine metabolism and phosphate transport systems (Figure 1E).

### 3.3. Fecal Metabolite Differences Between HFCR and LFCR Pigs

**Figure 2:**
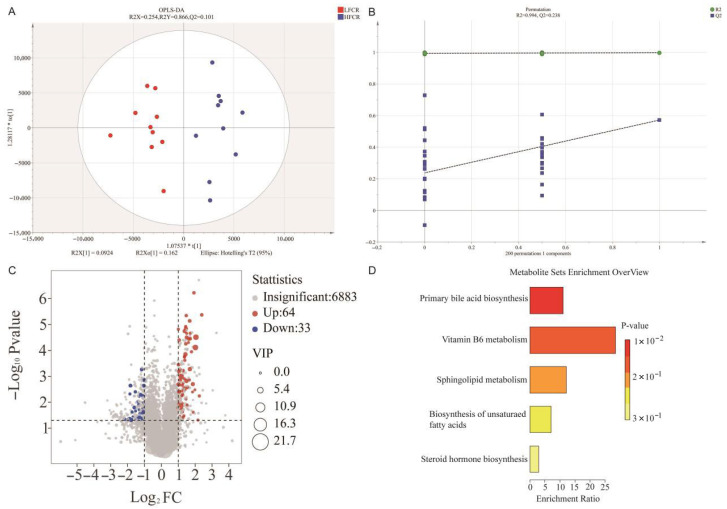
Fecal metabolomic profiling of pigs with divergent feed conversion ratios (FCR). (**A**) Orthogonal partial least squares discriminant analysis (OPLS-DA) showing separation between HFCR (blue) and LFCR (red) groups based on fecal metabolite profiles. (**B**) Permutation test (*n* = 200) validating the OPLS-DA model; R2 (green) indicates explanatory power and Q2 (blue) indicates predictive accuracy. (**C**) Volcano plot of differential metabolites; “Up” indicates enrichment in LFCR pigs and “Down” indicates enrichment in HFCR pigs. Variable importance in projection (VIP) scores reflect each metabolite’s contribution to group separation. (**D**) KEGG pathway enrichment analysis of significant metabolites, highlighting metabolic pathways associated with feed efficiency differences.

OPLS-DA modeling revealed clear separation between groups (R2X = 0.254, R2Y = 0.866, Q2 = 0.101; Figure 2A), with permutation testing confirming model robustness (Q2 = −0.376; Figure 2B). In total, 97 differential metabolites were identified (VIP > 1, *p* < 0.05, FC > 2 or < 0.5), including 64 upregulated and 33 downregulated in LFCR pigs (Figure 2C). KEGG pathway enrichment analysis indicated that differential metabolites were mainly involved in primary bile acid biosynthesis, vitamin B6 metabolism, sphingolipid metabolism, unsaturated fatty acid metabolism, and steroid hormone synthesis (Figure 2D). Representative metabolites are summarized in Table 3.

### 3.4. Correlation Analysis Among Phenotypes, Microbiota, and Metabolites

**Figure 3:**
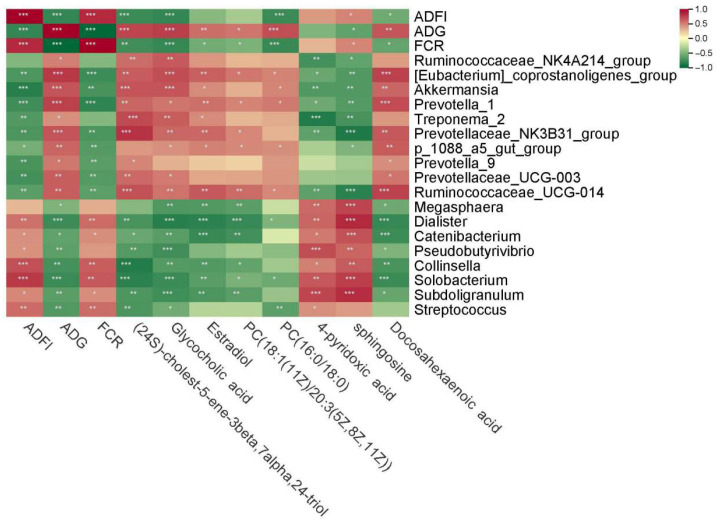
Spearman correlation heatmap of phenotypes, differential microbiota and differential metabolites. Red represents positive correlation and green represents negative correlation. Darker colors represent stronger associations. Significance levels: * *p* < 0.05, ** *p* < 0.01, *** *p* < 0.001.

Spearman correlation analysis (correlation coefficient > 0.6, *p* < 0.05) identified relationships among phenotypic traits, differential microbes, and metabolites (Figure 3). *Ruminococcus* and *Prevotella* were negatively correlated with FCR, while *Collinsella*, *Solobacterium*, *Subdoligranulum* and *Catenibacterium* showed significant positive correlations with FCR. Additionally, the *[Eubacterium]_coprostanogenes_group* and *Ruminococcaceae_NK4A214_group* were positively correlated with bile acid-related metabolites, while *Akkermansia* was positively correlated with estradiol.

## 4. Discussion

### 4.1. Differences in Fecal Microbiota Between High and Low Feed Conversion Efficiency Pigs

Alpha diversity indices are widely used to evaluate microbial richness and evenness. Previous studies have linked higher microbial diversity to improved pig growth performance. For example, Tan et al. reported significantly higher alpha diversity in the fecal microbiota of LFCR pigs, a finding consistent with our results [19]. Similarly, Metzler et al. observed that pigs with low RFI exhibited greater cecal microbial diversity [20]. In our study, LFCR pigs showed significantly elevated values for Richness, Chao1, and ACE indices compared to HFCR pigs, suggesting a more diverse and possibly more metabolically beneficial gut microbial community that may support enhanced nutrient utilization and overall health.

Gut microbes play a critical role in fermenting undigested dietary components, producing metabolites such as SCFAs and bile acids that exert various physiological effects. Notably, members of Prevotellaceae [21] and Ruminococcaceae [22] are key fiber-degrading taxa that contribute significantly to SCFA production. *Prevotella_1* specializes in breaking down hemicellulose or xylan, thereby supplying energy [23]. Similarly, *Prevotellaceae_UCG-003* has been noted for its role in intestinal regulation and its capacity to break down fibrous polysaccharides into SCFAs. *Prevotellaceae_NK3B31_group* is associated with increased acetic acid, propionic acid, and total SCFAs, particularly in weaned piglets [24]. Moreover, the *Ruminococcaceae_NK4A214_group* demonstrates increased abundance in the rumen of low RFI cattle [25], a finding paralleling our own observations. Research indicates that *Ruminococcaceae UCG-014* has the capability to produce butyric acid [26]. Additionally, *[Eubacterium]_coprostanogenes_group* contributes to SCFA production [27]. *Akkermansia*, recognized as a significant fecal biomarker linked with weight gain [28], shows that reduced Akkermansia abundance is directly associated with increased adiposity, insulin resistance, and dyslipidemia in obese mice [29]. Studies also highlight *Akkermansia*’s mucin-degrading capability and its role as an SCFA producer [30]. The enrichment of these SCFA-producing bacteria in LFCR pigs may enhance nutrient absorption and gut health, ultimately improving feed conversion efficiency.

Interestingly, some SCFA-producing taxa were also enriched in HFCR pigs. For example, *Prevotella_1* and *Prevotellaceae_UCG_003* were reported by Xue et al. to be more abundant in high RFI sheep [31]. *Pseudobutyrivibrio*, enriched in the distal colon, is positively associated with free fatty acid levels [32]. *Subdoligranulum* and *Megaspaera*, which convert lactate to SCFAs, were significantly enriched in the cecum of HFCR Landrace pigs [33]. However, *Subdoligranulum* has also been positively associated with depressive phenotypes in humans [34], suggesting that the physiological effects of SCFAs may be context-dependent and require further clarification.

Moreover, several bacteria enriched in HFCR pigs have been associated with adverse health outcomes. *Collinsella* has been linked to obesity, atherosclerosis [35], and hypercholesterolemia [36]. Lahti reported a significant positive correlation between *Collins* bacteria and TG (triglycerides) levels [37]. *Solobacterium*, part of the Erysipelotrichaceae family, is implicated in obesity, inflammatory bowel disease (IBD) [38], and colorectal cancer [39]. *Streptococcus*, a common swine pathogen and potential zoonotic agent, is a reservoir for antibiotic resistance genes [40]. The enrichment of these potentially pathogenic or dysbiotic bacteria in HFCR pigs may impair gut health and contribute to reduced feed efficiency.

### 4.2. Differential Fecal Metabolites Between High and Low Feed Conversion Efficiency Pigs

Bile acids are essential for lipid digestion, nutrient absorption, and energy metabolism. Their synthesis and transformation are closely linked to gut microbial activity and feed utilization efficiency in livestock [41]. In our study, the LFCR group exhibited significantly higher levels of bile acid-related metabolites, including (24S)Cholest-5-ene-3β,7α,24-triol and glycocholic acid. The former is a bile alcohol that arises during bile acid metabolism, while the latter is a conjugated bile acid with antibacterial properties. Bile alcohol can be seen as an intermediate and byproduct of the normal pathway in bile acid biosynthesis, playing a crucial role in food digestion and nutrient absorption [13]. Primary bile acids combine with glycine and taurine to form conjugated bile acids. Binding bile acids are influenced by microorganisms in the intestine to remove binding and exert their effects. Glycine bile acid is a conjugated bile acid with certain antibacterial properties [42]. The metabolic products associated with bile acids were notably higher in the LFCR group compared to the HFCR group, suggesting enhanced bile acid biosynthesis and transformation in pigs. This enhancement likely boosts the absorption of dietary fats and fat-soluble vitamins, leading to increased energy assimilation.

Unsaturated fatty acids, particularly omega-3 polyunsaturated fatty acids (PUFAs) like docosahexaenoic acid (DHA), possess multiple metabolic benefits, including anti-inflammatory effects [43], lipid-lowering activity [44], and cardiovascular protection [45]. PUFAs have also been shown to modulate fecal microbiota, notably increasing *Akkermansia* abundance, improving intestinal barrier integrity, and enhancing mucosal immunity [46]. Steroid hormones can affect the production of growth hormone by regulating endocrine factors related to growth [47]. As a steroid hormone, estrogen exerts pivotal regulatory control over reproductive tract and mammary gland development in sows [48]. The elevated levels of DHA and estradiol observed in LFCR pigs may contribute to superior growth performance and feed efficiency.

### 4.3. Integrated Microbiome-Metabolome Analysis Reveals Determinants of Feed Efficiency

Integrated correlation analysis revealed significant positive associations between bile acid–related metabolites (e.g., (24S)-Cholest-5-ene-3β,7α,24-triol, Glycocholic acid) and the bacterial taxa *[Eubacterium]_coprostanogenes_group* and *Ruminococcaceae_NK4A214_group*. Gut microbes are known to mediate bile acid dehydroxylation and transformation, converting primary to secondary bile acids [49]. Bile acids play a crucial role in bile acid metabolism and promote lipid digestion and absorption, regulate cholesterol metabolism, and stimulate bile secretion [50]. Taxa such as *Bacteroides*, *Clostridium*, *Escherichia*, and *Eubacterium* have demonstrated dehydroxylation effects [51]. Notably, *[Eubacterium]_coprostanogenes_group* has been reported to produce sterol derivatives [27], supporting its role in bile acid metabolism. Similarly, *Ruminococcaceae_NK4A214_group* has been shown to increase bile acid content when transplanted into mice, further supporting its contribution to improved fat and vitamin absorption, thereby enhancing feed conversion efficiency [52].

**Figure 4:**
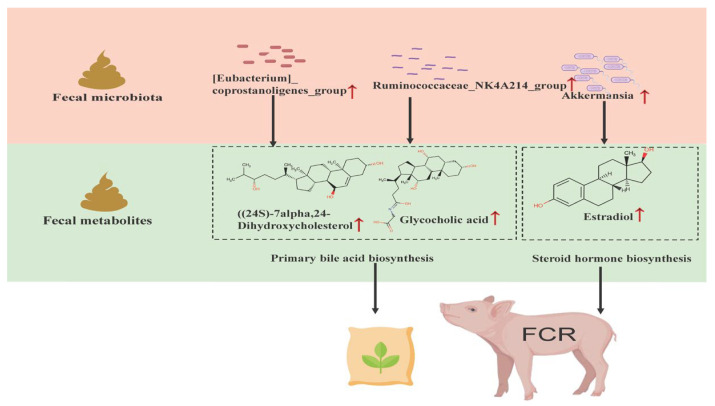
Proposed model illustrating how fecal microbiota modulate feed efficiency in pigs through bile acid metabolism and steroid hormone regulation. Arrows indicate significant correlations. Red represents positive associations.

Furthermore, a strong positive correlation was observed between *Akkermansia* and estradiol. Prior studies indicate that *Akkermansia* supplementation can reduce obesity, inflammation, and insulin resistance [29]. Estradiol treatment has also been shown to increase the abundance of *Akkermansia* in ovariectomized mice [53]. In women, oral administration of heat-killed *Akkermansia* led to significant reductions in fat mass [54]. These findings suggest that *Akkermansia* may act synergistically with estradiol to support growth and improve feed efficiency in pigs (Figure 4).

### 4.4. Limitations and Future Directions

This study is observational and cross-sectional with a relatively small sample size for the fecal microbiota and metabolomics analyses; therefore, the findings should be interpreted as associations rather than causal effects. Although animals were matched for environment, genetic background, and sex, residual confounding cannot be fully excluded. To improve generalizability, subsequent studies should expand the cohort and include independent populations. In addition, we did not perform functional validation for the specific microbes and metabolites identified here. Future work will incorporate fecal microbiota transplantation (FMT) to probe microbial functions and controlled pig feeding trials to test the functional roles of candidate metabolites.

## 5. Conclusions

This study revealed that pigs with LFCR harbor a more diverse fecal microbiota enriched in SCFA-producing bacteria, while HFCR pigs show increased levels of potentially pathogenic microbes. Metabolomic analysis identified differential enrichment of bile acid and steroid hormone-related metabolites. Notably, *Ruminococcaceae_NK4A214_group* and *[Eubacterium]_coprostanogenes_group* were associated with bile acid metabolism, and *Akkermansia* correlated with estradiol levels. Together, our results indicate that fecal microbiota and their metabolites are associated with feed efficiency. While causality remains to be established, the identified microbial and metabolic markers suggest actionable targets for precision breeding, probiotic supplementation, and dietary interventions in swine.

## Figures and Tables

**Table 1:** Growth performances of HFCR and LFCR pigs.

Trait	LFCR	HFCR	*p*-Values
ADFI (kg/day)	2.30 ± 0.05	2.69 ± 0.11	<0.001
ADG (kg/day)	1.24 ± 0.08	1.04 ± 0.04	<0.001
FCR	1.86 ± 0.14	2.56 ± 0.10	<0.001

**Table 2:** Alpha diversity indices of fecal microbiota in HFCR and LFCR pigs.

Index	LFCR	HFCR	*p*-Values
Richness	6415.00 ± 426.78	5690.30 ± 629.75	<0.01
Chao1	6813.65 ± 330.58	6141.44 ± 637.70	<0.01
ACE	6742.31 ± 336.16	6060.13 ± 633.08	<0.01
Shannon	7.91 ± 0.25	7.62 ± 0.23	0.05

**Table 3:** Representative differential metabolites between HFCR and LFCR pigs.

Compound	Mass	Formula	VIP	FC	*p*-Values	HMDB
PC(18:1(11Z)/20:3(5Z,8Z,11Z))	809.59	C_46_H_84_NO_8_P	2.80	2.73	<0.01	HMDB0060289
PC(16:0/18:0)	761.59	C_42_H_84_NO_8_P	PC(16:0/18:0)	2.37	<0.01	HMDB0000978
estradiol	272.17	C_18_H_24_O_2_	2.02	2.58	<0.01	HMDB0000151
4-pyridoxic acid	118.03	C_8_H_9_NO_4_	2.33	3.9	<0.01	HMDB0000202
(24S)-cholest-5-ene-3beta,7alpha,24-triol	418.34	C_27_H_46_O_3_	2.45	3.22	<0.01	HMDB0060136
glycocholic acid	465.31	C_26_H_43_NO_6_	1.81	2.08	<0.01	HMDB0000138
sphingosine	299.28	C_18_H_37_NO_2_	2.60	0.41	0.04	HMDB0000252
docosahexaenoic acid	328.24	C_22_H_32_O_2_	2.73	2.05	<0.001	HMDB0002183

Mass = relative molecular mass; Formula = molecular formula; VIP = variable importance in projection; FC = fold change (LFCR/HFCR); HMDB = Human Metabolome Database.

## Data Availability

The data of 16s rRNA sequencing in this study have been deposited in the NCBI Gene Expression Omnibus (GEO) under the accession number PRJNA1072006, and metabolomics have been deposited in the MetaboLights under the accession number MTBLS9500.

## Supplementary Materials

The following supporting information can be downloaded at https://www.mdpi.com/article/10.3390/ani15203026/s1: Figure S1: QC sample mass spectrum TIC overlay; Table S1: Base ration composition and nutrient levels; Table S2: Phenotypic traits of HRCR and LFCR pigs; Table S3: Phenotypic traits of 384 pigs; Table S4: Differential metabolites between HRCR and LFCR pigs.
