# Supplementary material for: Identification of Fecal Microbiota and Related Metabolites Associated with Feed Efficiency in DLY Pigs
Source: Animals (Basel). 2025 Oct 18;15(20):3026. doi: 10.3390/ani15203026 (PMC12562226; doi:10.3390/ani15203026)
Supplement: Supplementary file 1 [file animals-15-03026-s001.zip › animals-3894918-supplementary.pdf]

Supporting Information

Identification of Fecal Microbiota and Related Metabolites Associated with Feed Efficiency in DLY Pigs

Table S1. Base ration composition and nutrient levels

Material	Content (%)	Nutrient levels	Content (%)
Corn	57.00	Net energy (Kcal/Kg)	2280
Soybean meal 46%	8.40	Crude protein	14.00
Bran	12.00	Crude fiber	3.70
Corn DDGS	8.00	Crude ash	2.50
Brown rice	5.00	Calcium	0.35
Rice bran meal	6.70	Total phosphorus	0.50
Stone flour	0.60	Lysine	0.83
Montmorillonite	0.50	Methionine	0.24
Calcium hydrogen phosphate	0.30	Threonine	0.52
Lysine	0.50	Tryptophan	0.12
Threonine	0.10		
Methionine	0.02		
Sodium chloride	0.50		
Probiotic preparations	0.02		
Phytase	0.02		
Antioxidants	0.02		
Anti-mold agent	0.10		
Complex minerals	0.20		
Vitamin complex	0.02		
total	100.00		

Note: All nutrient levels are measured, except net energy (NE), which is calculated.

Table S2. Phenotypic traits of HRCR and LFCR pigs

Number	ADFI	ADG	FCR	Group
F26	2234.53	1355.33	1.65	LFCR
F27	2267.12	1362.21	1.66	LFCR
F28	2251.87	1308.89	1.72	LFCR
F29	2284.38	1287.61	1.77	LFCR
F30	2370.05	1242.32	1.91	LFCR
F31	2271.79	1165.39	1.95	LFCR
F32	2392.85	1213.43	1.97	LFCR
F33	2273.89	1144.59	1.99	LFCR
F34	2358.13	1169.92	2.02	LFCR
F35	2354.78	1164.50	2.02	LFCR
F36	2650.55	1063.27	2.49	HFCR
F37	2670.87	1067.48	2.50	HFCR
F38	2737.23	1093.78	2.50	HFCR
F39	2715.67	1077.77	2.52	HFCR
F40	2767.52	1097.86	2.52	HFCR
F41	2635.62	1044.24	2.52	HFCR
F42	2526.40	981.30	2.57	HFCR
F43	2793.02	1078.81	2.59	HFCR
F44	2507.35	967.80	2.59	HFCR
F45	2878.11	1017.72	2.83	HFCR

ADFI = average daily feed intake(g/day); ADG = average daily gain(g/day); FCR = ADFI/ADG. The LFCR group contains: F26-F35(n = 10). The HFCR group contains: F36-F45(n = 10).

Table S3. Phenotypic traits of 384 pigs

Number	ADFI	ADG	FCR
F26	2234.53	1355.33	1.65
F27	2267.12	1362.21	1.66
F28	2251.87	1308.89	1.72
F29	2284.38	1287.61	1.77
F30	2370.05	1242.32	1.91
F31	2271.79	1165.39	1.95
F32	2392.85	1213.43	1.97
F33	2273.89	1144.59	1.99
F34	2358.13	1169.92	2.02
F35	2354.78	1164.50	2.02
F46	2286.54	1127.81	2.03
F47	2188.09	1078.62	2.03
F48	2038.81	1003.84	2.03
F49	2148.94	1057.21	2.03
F50	2486.43	1223.22	2.03
F51	2387.42	1170.25	2.04
F52	2312.23	1132.91	2.04
F53	2141.54	1048.39	2.04
F54	2242.27	1093.36	2.05
F55	2204.42	1074.12	2.05
F56	2241.59	1089.64	2.06
F57	2019.72	981.34	2.06
F58	2098.52	1017.30	2.06
F59	2452.07	1186.93	2.07
F60	2314.35	1119.75	2.07
F61	2187.50	1058.33	2.07
F62	2519.39	1218.81	2.07
F63	2013.64	973.31	2.07
F64	2164.95	1044.55	2.07
F65	2397.55	1156.38	2.07
F66	1854.28	893.54	2.08
F67	2599.04	1252.29	2.08
F68	1993.81	959.83	2.08
F69	2122.92	1021.69	2.08
F70	2418.14	1163.45	2.08
F71	2341.48	1126.21	2.08
F72	2419.44	1163.70	2.08
F73	2825.83	1359.13	2.08
F74	2163.07	1039.06	2.08
F75	2362.14	1133.98	2.08
F76	2429.21	1165.87	2.08

F77	2260.37	1084.58	2.08
F78	2222.91	1065.19	2.09
F79	2388.71	1143.10	2.09
F80	2202.02	1053.23	2.09
F81	2257.33	1079.65	2.09
F82	2231.70	1066.95	2.09
F83	2249.80	1075.15	2.09
F84	2515.31	1201.15	2.09
F85	2789.01	1331.80	2.09
F86	2346.96	1120.24	2.10
F87	2325.78	1109.74	2.10
F88	2129.15	1015.86	2.10
F89	2224.46	1061.33	2.10
F90	2454.87	1170.25	2.10
F91	2205.58	1049.82	2.10
F92	2162.45	1029.21	2.10
F93	2275.97	1083.20	2.10
F94	2648.70	1258.90	2.10
F95	2102.53	998.90	2.10
F96	2339.63	1109.62	2.11
F97	2371.63	1124.34	2.11
F98	2523.44	1195.81	2.11
F99	2404.87	1139.04	2.11
F100	2326.36	1101.79	2.11
F101	2309.40	1092.93	2.11
F102	2363.44	1118.44	2.11
F103	2440.69	1151.88	2.12
F104	2192.11	1034.37	2.12
F105	2233.88	1054.05	2.12
F106	2642.68	1246.60	2.12
F107	2515.73	1186.40	2.12
F108	2286.32	1077.91	2.12
F109	2032.38	958.15	2.12
F110	2069.55	975.67	2.12
F111	2124.73	1001.47	2.12
F112	2213.01	1042.25	2.12
F113	2603.37	1225.93	2.12
F114	2689.32	1266.07	2.12
F115	2651.30	1248.09	2.12
F116	1838.89	865.60	2.12
F117	2143.80	1008.50	2.13
F118	2373.31	1116.35	2.13
F119	2188.66	1028.08	2.13

F120	1957.56	918.99	2.13
F121	2512.43	1179.27	2.13
F122	2178.43	1022.41	2.13
F123	2261.91	1060.65	2.13
F124	2311.08	1083.63	2.13
F125	2285.60	1071.05	2.13
F126	2352.69	1102.06	2.13
F127	2344.19	1097.31	2.14
F128	2489.65	1164.24	2.14
F129	2191.69	1024.53	2.14
F130	2254.92	1052.50	2.14
F131	2386.96	1114.13	2.14
F132	2429.03	1133.60	2.14
F133	2189.39	1021.53	2.14
F134	2506.56	1168.96	2.14
F135	2246.21	1046.87	2.15
F136	2027.28	944.63	2.15
F137	2645.58	1232.15	2.15
F138	2267.32	1055.40	2.15
F139	2378.05	1105.11	2.15
F140	2151.68	999.24	2.15
F141	2695.30	1251.15	2.15
F142	2061.51	956.78	2.15
F143	2310.77	1071.74	2.16
F144	2295.19	1064.30	2.16
F145	2708.43	1254.79	2.16
F146	2317.14	1073.26	2.16
F147	2500.20	1156.90	2.16
F148	2155.50	997.14	2.16
F149	2461.02	1138.24	2.16
F150	2312.69	1068.33	2.16
F151	2133.31	985.42	2.16
F152	2201.46	1016.67	2.17
F153	2439.45	1126.51	2.17
F154	2301.41	1062.76	2.17
F155	2450.26	1131.31	2.17
F156	2594.32	1197.18	2.17
F157	2194.14	1012.12	2.17
F158	2413.63	1113.01	2.17
F159	2062.42	950.60	2.17
F160	2030.70	935.50	2.17
F161	2388.58	1100.29	2.17
F162	2234.62	1029.27	2.17

F163	2469.23	1136.78	2.17
F164	2397.67	1103.42	2.17
F165	2475.60	1139.01	2.17
F166	2358.51	1084.55	2.17
F167	2274.30	1044.20	2.18
F168	2343.98	1075.89	2.18
F169	2266.26	1039.84	2.18
F170	2178.13	999.13	2.18
F171	2407.91	1103.42	2.18
F172	2154.16	986.36	2.18
F173	2528.81	1156.66	2.19
F174	2317.63	1059.99	2.19
F175	2317.73	1059.93	2.19
F176	2499.19	1142.70	2.19
F177	2462.76	1125.85	2.19
F178	2356.66	1077.19	2.19
F179	2356.20	1075.68	2.19
F180	2492.58	1136.64	2.19
F181	2940.71	1340.19	2.19
F182	2383.08	1085.30	2.20
F183	2230.77	1015.55	2.20
F184	2148.10	977.45	2.20
F185	2357.21	1072.29	2.20
F186	2299.58	1045.17	2.20
F187	2477.02	1125.36	2.20
F188	2320.27	1053.79	2.20
F189	2379.43	1079.72	2.20
F190	2305.39	1046.03	2.20
F191	2596.11	1177.90	2.20
F192	2152.69	976.64	2.20
F193	1965.14	890.68	2.21
F194	2525.93	1143.71	2.21
F195	2225.18	1006.40	2.21
F196	2371.15	1071.63	2.21
F197	2480.92	1121.15	2.21
F198	2404.85	1086.43	2.21
F199	2364.12	1067.97	2.21
F200	2096.60	946.14	2.22
F201	2661.00	1200.74	2.22
F202	2410.66	1086.66	2.22
F203	2556.90	1151.07	2.22
F204	2431.67	1094.49	2.22
F205	2576.61	1159.41	2.22

F206	2311.43	1040.02	2.22
F207	2487.78	1119.37	2.22
F208	2371.59	1066.91	2.22
F209	2526.73	1136.62	2.22
F210	2330.94	1048.04	2.22
F211	2579.17	1158.78	2.23
F212	2313.82	1038.14	2.23
F213	2142.09	960.97	2.23
F214	2356.87	1057.19	2.23
F215	2263.33	1014.87	2.23
F216	2777.70	1245.11	2.23
F217	2502.90	1120.92	2.23
F218	2333.04	1044.79	2.23
F219	2385.05	1068.06	2.23
F220	2615.32	1170.68	2.23
F221	2505.08	1121.30	2.23
F222	2418.52	1082.21	2.23
F223	2091.47	935.67	2.24
F224	2136.62	955.02	2.24
F225	2568.73	1147.96	2.24
F226	2624.37	1172.73	2.24
F227	2287.66	1022.00	2.24
F228	2354.71	1051.40	2.24
F229	2426.77	1083.41	2.24
F230	2334.89	1041.25	2.24
F231	2162.81	964.35	2.24
F232	2331.95	1039.66	2.24
F233	2403.83	1071.54	2.24
F234	2502.32	1114.69	2.24
F235	2446.19	1089.33	2.25
F236	2245.42	999.78	2.25
F237	2232.18	993.80	2.25
F238	2003.58	891.57	2.25
F239	2256.82	1004.17	2.25
F240	2853.77	1269.30	2.25
F241	2414.35	1073.73	2.25
F242	2629.86	1169.06	2.25
F243	2146.92	953.99	2.25
F244	2328.39	1033.96	2.25
F245	2152.29	955.38	2.25
F246	2655.08	1178.53	2.25
F247	2275.06	1009.83	2.25
F248	2577.92	1143.83	2.25

F249	2320.81	1029.36	2.25
F250	2452.22	1087.15	2.26
F251	2304.48	1021.31	2.26
F252	2484.55	1100.84	2.26
F253	2168.27	960.66	2.26
F254	2395.39	1061.05	2.26
F255	3172.55	1405.28	2.26
F256	2685.06	1188.96	2.26
F257	2267.81	1003.53	2.26
F258	1942.28	859.40	2.26
F259	2355.83	1042.28	2.26
F260	2018.81	892.78	2.26
F261	2263.62	1000.90	2.26
F262	2226.61	984.51	2.26
F263	2995.26	1323.43	2.26
F264	2263.69	999.79	2.26
F265	2253.41	993.94	2.27
F266	2522.58	1112.02	2.27
F267	2453.76	1081.35	2.27
F268	2252.72	992.53	2.27
F269	2540.08	1119.01	2.27
F270	2402.94	1057.68	2.27
F271	2394.90	1053.79	2.27
F272	2615.33	1150.08	2.27
F273	2539.91	1115.99	2.28
F274	2314.02	1016.01	2.28
F275	2384.09	1046.37	2.28
F276	2630.03	1154.30	2.28
F277	2522.86	1106.54	2.28
F278	2083.63	913.74	2.28
F279	2547.68	1116.50	2.28
F280	2375.44	1041.01	2.28
F281	2177.63	954.06	2.28
F282	2370.89	1038.61	2.28
F283	2373.11	1038.67	2.28
F284	2467.59	1079.91	2.29
F285	2513.14	1098.58	2.29
F286	2529.82	1105.42	2.29
F287	2720.80	1187.58	2.29
F288	2621.84	1143.05	2.29
F289	2268.64	988.91	2.29
F290	2447.50	1066.60	2.29
F291	2522.31	1098.88	2.30

F292	2560.12	1114.97	2.30
F293	2227.61	969.13	2.30
F294	1934.74	841.68	2.30
F295	2441.02	1060.92	2.30
F296	2366.87	1027.02	2.30
F297	2381.48	1033.05	2.31
F298	2742.19	1189.47	2.31
F299	2493.35	1081.52	2.31
F300	2309.89	1001.00	2.31
F301	2561.00	1108.95	2.31
F302	2273.33	984.24	2.31
F303	2273.88	983.45	2.31
F304	2755.51	1191.35	2.31
F305	2539.07	1097.59	2.31
F306	2216.15	957.26	2.32
F307	2601.24	1123.30	2.32
F308	2316.51	1000.30	2.32
F309	2860.60	1234.81	2.32
F310	2380.87	1027.62	2.32
F311	2459.61	1061.41	2.32
F312	2538.62	1095.42	2.32
F313	2452.69	1058.08	2.32
F314	2749.91	1183.15	2.32
F315	2814.86	1209.63	2.33
F316	2288.16	983.27	2.33
F317	2555.96	1098.06	2.33
F318	2676.24	1148.99	2.33
F319	2338.33	1003.31	2.33
F320	2587.69	1109.32	2.33
F321	2238.81	959.60	2.33
F322	2490.52	1067.33	2.33
F323	2504.17	1073.11	2.33
F324	2194.82	940.44	2.33
F325	2557.99	1095.83	2.33
F326	2202.60	943.48	2.33
F327	2303.33	986.21	2.34
F328	1857.60	795.25	2.34
F329	2369.73	1013.41	2.34
F330	2634.66	1125.89	2.34
F331	2342.70	1000.80	2.34
F332	2643.57	1129.31	2.34
F333	2386.98	1018.57	2.34
F334	2426.03	1034.53	2.35

F335	2383.74	1016.08	2.35
F336	2524.23	1075.65	2.35
F337	2428.50	1033.35	2.35
F338	2423.95	1030.02	2.35
F339	2576.43	1091.55	2.36
F340	2403.88	1017.52	2.36
F341	2318.30	980.73	2.36
F342	2572.89	1088.23	2.36
F343	2292.36	968.55	2.37
F344	2830.51	1194.99	2.37
F345	2212.25	933.01	2.37
F346	2174.22	915.71	2.37
F347	2304.85	970.39	2.38
F348	2659.45	1119.28	2.38
F349	2502.19	1053.04	2.38
F350	2302.98	968.85	2.38
F351	2356.75	990.96	2.38
F352	2636.00	1108.32	2.38
F353	2552.04	1072.82	2.38
F354	2564.28	1077.82	2.38
F355	2557.25	1074.64	2.38
F356	2613.32	1098.12	2.38
F357	2407.34	1011.22	2.38
F358	2492.81	1046.77	2.38
F359	2003.36	840.46	2.38
F360	2501.45	1048.58	2.39
F361	2519.21	1055.34	2.39
F362	2598.84	1086.12	2.39
F363	2578.70	1077.42	2.39
F364	2301.25	961.44	2.39
F365	2352.06	982.48	2.39
F366	2697.42	1125.32	2.40
F367	2446.84	1020.73	2.40
F368	2267.01	943.50	2.40
F369	2736.04	1137.48	2.41
F370	2356.07	979.09	2.41
F371	2505.03	1040.81	2.41
F372	2509.40	1041.00	2.41
F373	2726.10	1130.33	2.41
F374	2841.75	1177.64	2.41
F375	2141.97	887.55	2.41
F376	2619.41	1085.34	2.41
F377	2258.73	934.88	2.42

F378	2531.32	1047.42	2.42
F379	2708.59	1120.75	2.42
F380	2367.36	978.59	2.42
F381	2643.49	1092.20	2.42
F382	2278.33	940.45	2.42
F383	2751.29	1134.12	2.43
F384	2485.12	1023.98	2.43
F385	2617.05	1076.13	2.43
F386	2315.12	950.06	2.44
F387	2700.64	1108.21	2.44
F388	2629.03	1076.51	2.44
F389	2383.03	974.34	2.45
F390	2493.00	1018.68	2.45
F391	2255.15	919.60	2.45
F392	2646.69	1078.97	2.45
F393	2476.82	1009.71	2.45
F394	2751.20	1120.60	2.46
F395	2421.18	985.65	2.46
F396	2706.37	1101.01	2.46
F397	2425.93	983.98	2.47
F398	2392.58	970.22	2.47
F399	2430.85	985.04	2.47
F400	2351.52	951.53	2.47
F401	2302.08	931.46	2.47
F402	2582.98	1044.73	2.47
F403	2299.06	929.50	2.47
F404	2466.64	996.70	2.47
F405	2273.81	915.85	2.48
F406	2239.97	901.29	2.49
F407	2778.01	1117.11	2.49
F408	2075.98	833.96	2.49
F409	2317.23	930.87	2.49
F36	2650.55	1063.27	2.49
F37	2670.87	1067.48	2.50
F38	2737.23	1093.78	2.50
F39	2715.67	1077.77	2.52
F40	2767.52	1097.86	2.52
F41	2635.62	1044.24	2.52
F42	2526.40	981.30	2.57
F43	2793.02	1078.81	2.59
F44	2507.35	967.80	2.59
F45	2878.11	1017.72	2.83

Table S4. Differential metabolites between HRCR and LFCR pigs

ID	HMDB
4-Pyridoxic acid	HMDB0000017
Dihydroxyphenylalanine	HMDB0000181
Methylmalonic acid	HMDB0000202
Oxoglutaric acid	HMDB0000208
3-Hydroxymethylglutaric acid	HMDB0000355
Hexanoylglycine	HMDB0000701
Xanthurenic acid	HMDB0000881
L-fucopyranose 1-phosphate	HMDB0001265
Cyclic guanosine monophosphate	HMDB0001314
(3R)-3-hydroxy-3-methyl-5-(phosphonooxy)pentanoic acid	HMDB0001343
Ribonolactone	HMDB0001900
Warfarin	HMDB0001935
5beta-Cholestane-3alpha,7alpha,12alpha,23-tetrol	HMDB0001968
Nervonic acid	HMDB0002368
Glycerophosphoric acid	HMDB0002520
Cyanidin	HMDB0002708
Chlorogenic Acid	HMDB0003164
Nicotinuric acid	HMDB0003269
Gentisate aldehyde	HMDB0004062
Leucodopachrome	HMDB0004067
9alpha,15S-Dihydroxy-11-oxothromba-5Z,13E-dien-1-oic acid	HMDB0004242
3-Oxohexadecanoic acid	HMDB0010733
3-Guanidinopropionic acid	HMDB0013222
Aspartylcysteine	HMDB0028750
(1R,3aS,5aR,5bR,7aR,9R,10R,11aR,11bR,13aR,13bR)-10-(3,4-dihydroxybenzoyl)oxy-9-hydroxy-5a,5b,8,8,11a-pentamethyl-1-prop-1-en-2-yl-1,2,3,4,5,6,7,7a,9,10,11,11b,12,13,13a,13b-hexadecahydrocyclopenta[a]chrysene-3a-carboxylic acid	HMDB0029779
(+)-Calycanthidine	HMDB0030281
3-(1,1-Dimethyl-2-propenyl)-8-(3-methyl-2-butenyl)xanthyletin	HMDB0030730
Rhein	HMDB0032876
Benzofuran	HMDB0032929
Daidzin	HMDB0033991
Estragole	HMDB0034121
Withaperuvin F	HMDB0034400
Amygdalin	HMDB0035030
Camellenodiol	HMDB0035735
(3R)-3-acetyloxy-3-[(1R,2R,5R,6R,7R,10S,11R,14S)-11-(furan-3-yl)-5-(2-hydroxypropan-2-yl)-2,6,10-trimethyl-3,13-dioxo-12,15-dioxatetracyclo[8.5.0.01,14.02,7]pentadecan-6-yl]propanoic acid	HMDB0035771
7-O-Acetylaustroinulin	HMDB0036804
Ethanethioic acid, S-[1-methyl-1-[(1R,4R)-4-methyl-2-oxocyclohexyl]ethyl] ester	HMDB0037383

Ganoderiol G	HMDB0037780
3-Hydroxy-10'-apo-b,y-carotenal	HMDB0039019
Digalacturonate	HMDB0039721
5-(12,15-Heneicosadienyl)-1,3-benzenediol	HMDB0039871
Lactitol	HMDB0040937
(3b,5a,25R)-3-Hydroxyspirostan-6-one 3-[2-acetylarabinosyl-(1->6)-glucoside]	HMDB0041185
beta-Casomorphin(1-6)	HMDB0060168
Quinoline-4,8-diol	HMDB0060289
Carboxyphosphamide	HMDB0060449
2-Propylglutaric acid	HMDB0060684
1-Heptadecanoyl-sn-glycero-3-phosphoethanolamine	HMDB0061691
3-Methylpentane	HMDB0061885
[(2S)-3-hydroxy-2-(12-methyltetradecanoyloxy)propyl] 20-methylhenicosanoate	HMDB0094447
5-[6-(2,4-dihydroxyphenyl)-2-(2,6-dihydroxyphenyl)-4-methylcyclohex-3-ene-1-carbonyl]-3-[(1E)-3-methylbut-1-en-1-yl]benzene-1,2,4-triol	HMDB0126495
2-[(2-{[5,7-dihydroxy-2-(4-hydroxyphenyl)-4-oxo-4H-chromen-3-yl]oxy}-4,5-dihydroxy-6-(hydroxymethyl)oxan-3-yl)oxy]-4,5-dihydroxy-6-(hydroxymethyl)oxan-3-yl 3-(4-hydroxy-3,5-dimethoxyphenyl)prop-2-enoate	HMDB0127489
Scutellarein	HMDB0128587
5-Hydroxy-1H-indole-3-carboxylic acid	HMDB0134931
2,8-Quinolinediol	HMDB0240311
5-(1,1-Dimethylheptyl)-2-[5-hydroxy-2-(3-hydroxypropyl)cyclohexyl]phenol	HMDB0247502
Azinphos-methyl	HMDB0248807
2-[(4-{2-[(4-Cyclohexylbutyl)(cyclohexylcarbamoyl)amino]ethyl}phenyl)sulfanyl]-2-methylpropanoic acid	HMDB0252992
Lonidamine	HMDB0254158
Sphingosylphosphorylcholine	HMDB0258420
Difloxacin	HMDB0251259
Auda	HMDB0248721
Acetochlor	HMDB0247910
4-Acetamidobenzoic acid	HMDB0246328
1,5-Isoquinolinediol	HMDB0244237
Tromethamine	HMDB0240288
(24S)-Cholest-5-ene-3beta,7alpha,24-triol	HMDB0060136
Bufotenin	HMDB0041842
3-Hydroxy-8'-apo-epsilon-caroten-8'-al	HMDB0036885
(23S,24S)-17,23-Epoxy-24,29-dihydroxy-27-norlanost-8-ene-3,15-dione	HMDB0035843
Vitisifuran A	HMDB0034785
3-Methyl-1-butylamine	HMDB0031659
C.I. Natural Red 20	HMDB0030579
beta-Tocotrienol	HMDB0030554
Salicylamide	HMDB0015687
Acepromazine	HMDB0015552

Budesonide	HMDB0015353
Imatinib	HMDB0014757
Profenamine	HMDB0014536
Lorazepam	HMDB0014332
2-Hydroxyhexadecanoylcarnitine	HMDB0013337
3beta-Hydroxy-4beta-methyl-5alpha-cholest-7-ene-4alpha-carboxylic acid	HMDB0011662
N-Methylphthalimide	HMDB0004284
4-(2-Amino-3-hydroxyphenyl)-2,4-dioxobutanoic acid	HMDB0004083
Indole-3-carboxylic acid	HMDB0003320
Syringic acid	HMDB0002085
Clotrimazole	HMDB0001922
Aminohippuric acid	HMDB0001867
Tetrahydrodeoxycorticosterone	HMDB0000879
Docosanamide	HMDB0000583
5-hydroxy-L-tryptophan	HMDB0000472
24,25-Dihydroxycholecalciferol	HMDB0000430
Estradiol	HMDB0000151

A total of 93 differential metabolites with HMDB numbers were screened using VIP > 1, $p < 0.05$, FC > 2, or FC < 0.5 conditions. These differential metabolites will be further analyzed for KEGG enrichment pathways, with a focus on discussing metabolites in the KEGG pathway.

(A) Positive_QC_TIC

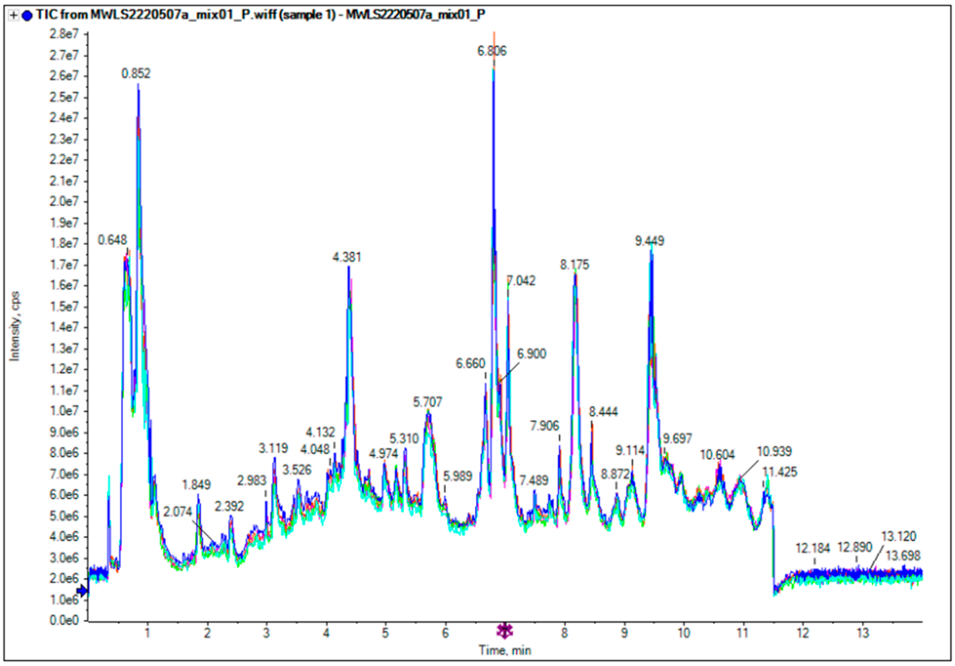

(B) Negative_QC_TIC

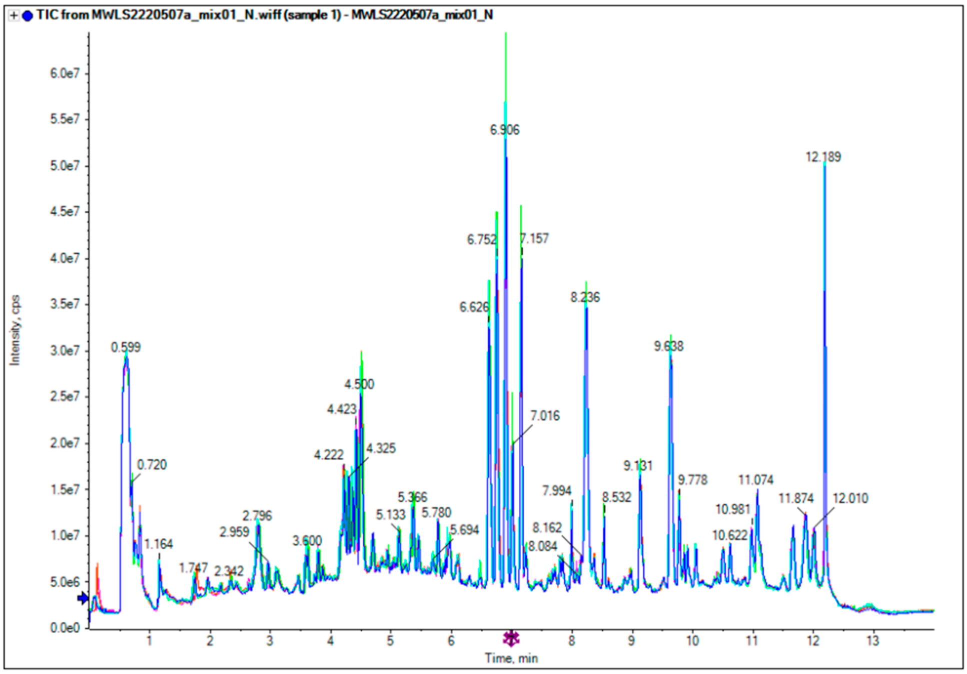

Figure S1. QC sample mass spectrum TIC overlay. **(A)** TIC diagram in positive ion mode. **(B)** TIC diagram in negative ion mode. The results show that the curve overlap of total ion current detected by metabolites is high, that is, the retention time and peak intensity are consistent, indicating that the signal stability is good when the same sample is detected at different times by mass spectrometry. The high stability of the instrument provides an important guarantee for data repeatability and improvement.
